# Perioperative use of ketamine infusion versus dexmedetomidine infusion for analgesia in obese patients undergoing bariatric surgery: a double-blinded three-armed randomized controlled trial

**DOI:** 10.1186/s12871-023-02059-3

**Published:** 2023-04-01

**Authors:** Belal Nabil Mahfouz Khalil, Maha Sadek Hussein Elderh, Mohamed Abdel Rasoul Khaja, Ahmed Nagah El-Shaer, Bahaa El-Din Ewees Hassan Ali, Mohamed Osman Awad Taeimah

**Affiliations:** 1 Department of Anesthesiology, Intensive Care and Pain Management, Faculty of Medicine, Ain Shams University, Cairo, Egypt; 2 Department of Anesthesiology and Intensive Care, Jahra Hospital, Ministry of Health, Jahra, Kuwait

**Keywords:** Obesity, Bariatric surgery, Ketamine, Dexmedetomidine, Analgesia, PONV

## Abstract

**Background:**

Bariatric surgery depends on the development of novel anesthetic techniques to reduce the incidence of complications and improve postoperative outcomes. Ketamine and dexmedetomidine have been used for perioperative analgesia and we hypothesized that they would decrease postoperative morphine requirements. The objective of this trial is to study whether choice of ketamine or dexmedetomidine infusion would affect postoperative total morphine consumption.

**Methods:**

Ninety patients were equally randomized into three groups. The ketamine group received a bolus dose (0.3 mg/kg) of ketamine over 10 min, followed by an infusion of the same drug (0.3 mg/kg/h). The dexmedetomidine group received a bolus dose (0.5 mcg/kg) of dexmedetomidine over 10 min, followed by an infusion of this drug (0.5 mg/kg/h). The control group received a saline infusion. All infusions were given till 10 min before the end of surgeries. Intraoperative fentanyl was given when patient developed hypertension and tachycardia despite adequate anesthesia and muscle relaxation. Postoperative pain was managed by a rescue dose of 4 mg of IV morphine, with a minimum interval of 6 h between morphine doses if the numerical rating scale (NRS) score was ≥ 4. The primary outcome was the total morphine dose, and the secondary outcomes were intraoperative fentanyl requirement, time to extubation, postoperative nausea and vomiting (PONV), NRS scores, and modified observer’s agitation/sedation scale (MOASS) scores.

**Results:**

Compared with ketamine, dexmedetomidine decreased the need for fentanyl intraoperatively (160 ± 42 µg), shortened the time to extubation (3 ± 1 min), and improved MOASS and PONV scores. In turn, ketamine decreased postoperative NRS scores and the need for morphine (3 ± 3 mg).

**Conclusions:**

Dexmedetomidine treatment was associated with lower fentanyl doses, a shorter time to extubation, and better MOASS and PONV scores. Ketamine treatment was associated with significantly lower NRS scores and morphine doses. These results indicated that dexmedetomidine effectively decreased intraoperative fentanyl requirement and the time to extubation, while ketamine decreased the need for morphine.

**Trial registration:**

This trail was registered on the clinicaltrials.gov registry (NCT04576975) on October 6, 2020.

**Supplementary Information:**

The online version contains supplementary material available at 10.1186/s12871-023-02059-3.

## Background

Surgical interventions aimed towards treating obesity are known as bariatric surgeries. There has been a drastic increase in the use of bariatric surgery for patients with medically complicated obesity who have difficulty losing weight by other methods [1].

Advances in anesthetic techniques have improved surgical procedures and clinical outcomes [2]. However, anesthesia can delay the recovery of obese patients with a high prevalence of respiratory conditions and sleep disorders [3]. In addition, the regular perioperative use of opiates in the management of bariatric surgeries has led to side effects, such as sedation, postoperative nausea and vomiting (PONV), respiratory depression, and reduced gastrointestinal motility [4], and these side effects increase the risk of developing cardiac and respiratory complications [5].

Judicious use of anesthetics is necessary because these drugs increase the risk of complications. Conversely, decreased use of opiates may result in postoperative pain, slowing postoperative recovery [6]. Therefore, we need to minimize opioid use while administering other drugs with analgesic and opioid-sparing effects [7].

Ketamine is an N-methyl-D-aspartate antagonist with analgesic and anti-hyperalgesic properties at low doses [8]. Ketamine prevents the development of opioid tolerance by minimizing the use of opioids while reducing postoperative pain, ultimately decreasing opioid-related postoperative morbidity [9].

Dexmedetomidine is a highly selective α_2_ adrenoceptor agonist used as an adjuvant analgesic in the perioperative period [10]. This drug improves hemodynamic stability and reduces the stress induced by intubation because of its central sympatholytic action [11]. Furthermore, dexmedetomidine decreases the need for opioids and anesthetics, providing additional benefits for obese patients [12].

This study compared the analgesic effect of ketamine and dexmedetomidine during bariatric surgery on postoperative total morphine consumption. We hypothesized that ketamine and dexmedetomidine would decrease postoperative morphine requirements for patients undergoing bariatric surgery.

## Materials and methods

This prospective, randomized, double-blinded, three-armed, controlled trial was registered at clinicaltrial.gov (NCT04576975) on October 6, 2020, and has been reported according to the CONSORT guidelines. The study was approved by the Research Ethics Committee of the Faculty of Medicine of Ain Shams University (FMASU MD 108 /2020) on June 14, 2020. All procedures were conducted in accordance with the tenets of the 2013 Declaration of Helsinki.

Based on a previous study [13], we anticipated the mean morphine consumption over 24 h to be 21.09 ± 12.88 in the control group. The study required a minimum of 22 patients per group to detect an effect size of 0.4 for mean morphine consumption in each group, with a power and type I error of 0.8 and 0.05, respectively, and to detect a difference of 40% in morphine consumption. The sample size was increased to 30 patients per group to compensate for the potential loss to follow-up, failure rate, and skewness.

After ethical approval, patients who were scheduled for elective laparoscopic sleeve gastrectomy were interviewed by a team member. The inclusion criteria were patients aged 21 to 45 years of both sexes, with a body mass index (BMI) > 35 kg/m^2^ and American Society of Anesthesiologists physical status class II or III. The exclusion criteria were patients with a history of hypersensitivity to dexmedetomidine or ketamine, a history of substance abuse (benzodiazepines) or chronic opioid use, psychiatric disorders, seizures, uncontrolled hypertension (HTN) (systolic ≥ 140 mmHg or diastolic ≥ 90 mmHg) or heart block, and uncontrolled diabetes mellitus (DM) (HbA1c ≥ 8.5%). Informed consent was obtained from patients who met the inclusion criteria. Patients were randomly allocated to one of three groups using computer-generated random numbers (Microsoft Excel 365). All syringes were prepared in a pharmacy and then coded and labeled. Infusions were performed using an infusion pump (CareFusion Alaris CC MKIII®, Alaris Medical Systems, Hampshire, UK). Infusion rates were measured by an independent pharmacist who was not associated with the study. Investigators were blinded to group assignment and drug coding. Independent anesthesiologists, who were not blinded, assessed the patients intraoperatively during the course of the study.

Baseline data on age, sex, BMI, and type of surgery were obtained. All tests (fasting blood glucose, HbA1c, thyroid profile, complete blood count, kidney and liver function tests, chest radiography, and electrocardiography) were performed according to our hospital’s protocols and reviewed before the day of operation. Upon entering the pre-anesthesia unit, the patients were premedicated with intravenous (IV) midazolam (20 µg/kg total body weight [TBW]) and IV atropine (0.015 mg/kg lean body weight [LBW]). Patients were randomly divided into the following three groups:

- Ketamine group received a bolus dose of ketamine (Sterop, Brussels, Belgium) (0.3 mg/kg ideal body weight [IBW]) diluted with 0.9% normal saline (NS) and infused over a 10-min period using a 20-mL syringe before induction, followed by an infusion of ketamine (10 mg/mL, 500 mg vial diluted in a 50-cc syringe) at a rate of 0.3 mg/kg/h until 10 min before the end of the surgery.
- Dexmedetomidine group received a bolus dose of dexmedetomidine (Precedex®; Hospira, Birmingham, AL, USA) (0.5 µg/kg IBW) diluted with 0.9% NS and infused over a 10-min period using a 20-mL syringe before induction, followed by an infusion of dexmedetomidine (4 µg/mL, 200 µg vial diluted in a 50-cc syringe) at a rate of 0.5 µg/kg/h until 10 min before the end of the surgery.
- Control group received a bolus dose of 0.9% NS over a 10-min period using a 20-mL syringe infused before induction, followed by an infusion of 0.9% NS (50 mL in a 50-cc syringe).

General anesthesia was induced with intravenous lidocaine (0.5 mg/kg IBW), propofol (1.5 mg/kg LBW), fentanyl (1.0 µg/kg TBW), and rocuronium (1.1 mg/kg IBW) for rapid-sequence endotracheal intubation. There were no cases of failed intubation in our cohort. Rocuronium was given every 30 min, and its effect was assessed by neuromuscular blockade monitoring keeping the train-of-four ratio between 0 and 1. General anesthesia was maintained with sevoflurane in an oxygen and air mixture guided by a bispectral index score between 40 and 60. Patients were mechanically ventilated with the maintenance of normocapnia (end-tidal CO_2_: 35–40 mmHg). Intraoperative vital data were recorded every 5 min.

If hypotension (defined as a mean arterial pressure [MAP] < 20% of baseline shown by two consecutive readings within 5 min, not responding to a 1% decrease in the inspired sevoflurane concentration) occurred, a crystalloid fluid incremental bolus (20 mL/kg IBW over 20 min) was administered, and then ephedrine was administered in 6-mg increments. For bradycardia (defined as a heart rate [HR] below 60 mmHg for ≥ 1 min), atropine 0.5 mg was administered as determined by the anesthesiologist.

Intraoperative pain was defined as the development of HTN and tachycardia despite adequate anesthesia and muscle relaxation. HTN was defined as an MAP of > 20% of baseline on two consecutive readings within 5 min. Tachycardia was defined as an HR of > 20% of baseline for 5 min. A rescue dose of fentanyl was administered in increments of 0.5 µg/kg TBW. The intraoperative total fentanyl dose for each patient was calculated.

Upon awakening, the patient received sugammadex (2 mg/kg) with a repeated dose of 4 mg/kg after 5 min if needed for muscle relaxation. A record of “time to extubation,” which was defined as the time taken for tracheal extubation after the discontinuation of anesthesia, was made for every patient.

All patients were monitored postoperatively in the post-anesthesia care unit and ward. Sedation was assessed using the modified observer’s assessment of alertness/sedation scale (MOASS) (Table 1). Scores were recorded at 0, 10, 30, and 60 min from admission to post anesthesia care unit (PACU).

**Table 1:** Modified observer’s assessment of alertness/sedation scale

Response	Score
Agitated	6
Responds readily to name spoken in a normal tone (alert)	5
Lethargic response to name spoken in a normal tone	4
Responds only after the name is called loudly or repeatedly	3
Responds only after mild prodding or shaking	2
Does not respond to mild prodding or shaking	1
Does not respond to deep stimulus	0

Postoperative pain was assessed using the numerical rating scale (NRS), with scores varying from 0 (no pain) to 10 (worst pain). The patients were informed preoperatively about the use and values of the NRS. The NRS score was recorded at 0, 30, and 60 min and 2, 6, 12, and 24 h after surgery.

All patients received 1 gm of IV paracetamol (Perfalgan®; Bristol-Myers Squibb, Munich, Germany) every 6 h for 24 h postoperatively. In addition, a rescue dose of 4 mg of IV morphine with a minimum 6-h interval was administered between doses if the NRS score was ≥ 4, if needed. The total doses of morphine were calculated.

All patients were assessed for PONV using the PONV intensity scale (Table 2). Assessments were performed at 0, 6, 12, and 24 h after recovery from general anesthesia. Four milligrams of ondansetron (Zofran®; GlaxoSmithKline, Rockville, ML) were administered in cases of severe nausea and repeated after 30 min if necessary.

**Table 2:** Postoperative nausea and vomiting intensity scale

Have you vomited or had dry retching? *	No	0
	Once or twice	1
	Thrice or more	50
Have you experienced a feeling of nausea (“an unsettled feeling in the stomach and slight urge to vomit”)? If yes, has nausea interfered with activities of daily living, such as getting out of bed, moving in bed, walking, eating, and drinking?	No	0
	Sometimes	1
	Often or most of the times	2
	All the time	25
How has your nausea been mostly?	Varying, comes and goes	1
	Constant, almost always present	2
What was the duration of your feeling of nausea (in hours [whole or fraction])?	____: ____ h	

*Count distinct episodes: several vomiting or retching events occurring over a short period (e.g., 5 min) should be counted as one vomiting or retching episode; otherwise, these events should be counted separately [14]

Postoperative complications (airway obstruction, hypoxia, severe nausea, respiratory depression, and vomiting) were recorded.

Data were coded, tabulated, and analyzed using SPSS software version 16.0 (SPSS Inc., Chicago, USA) for Windows. Continuous variables were presented as mean (standard deviation) or median (Q3-Q1) and were compared using one-way analysis of variance or the Kruskal-Wallis test. Categorical variables were presented as frequency (%) and were compared using the chi-square test. Multiple pairwise comparisons were performed using the Tukey honestly significant difference test, Mann–Whitney test, or chi-square test. A P-value of less than 0.05 was considered statistically significant, and Bonferroni correction was applied when necessary.

## Primary and secondary outcomes

The primary outcome was the need for analgesics postoperatively in the form of total morphine consumption. The secondary outcomes were the need for analgesics intraoperatively, time to extubation, postoperative sedation scores, NRS scores, and PONV scores.

## Results

**Fig. 1:**
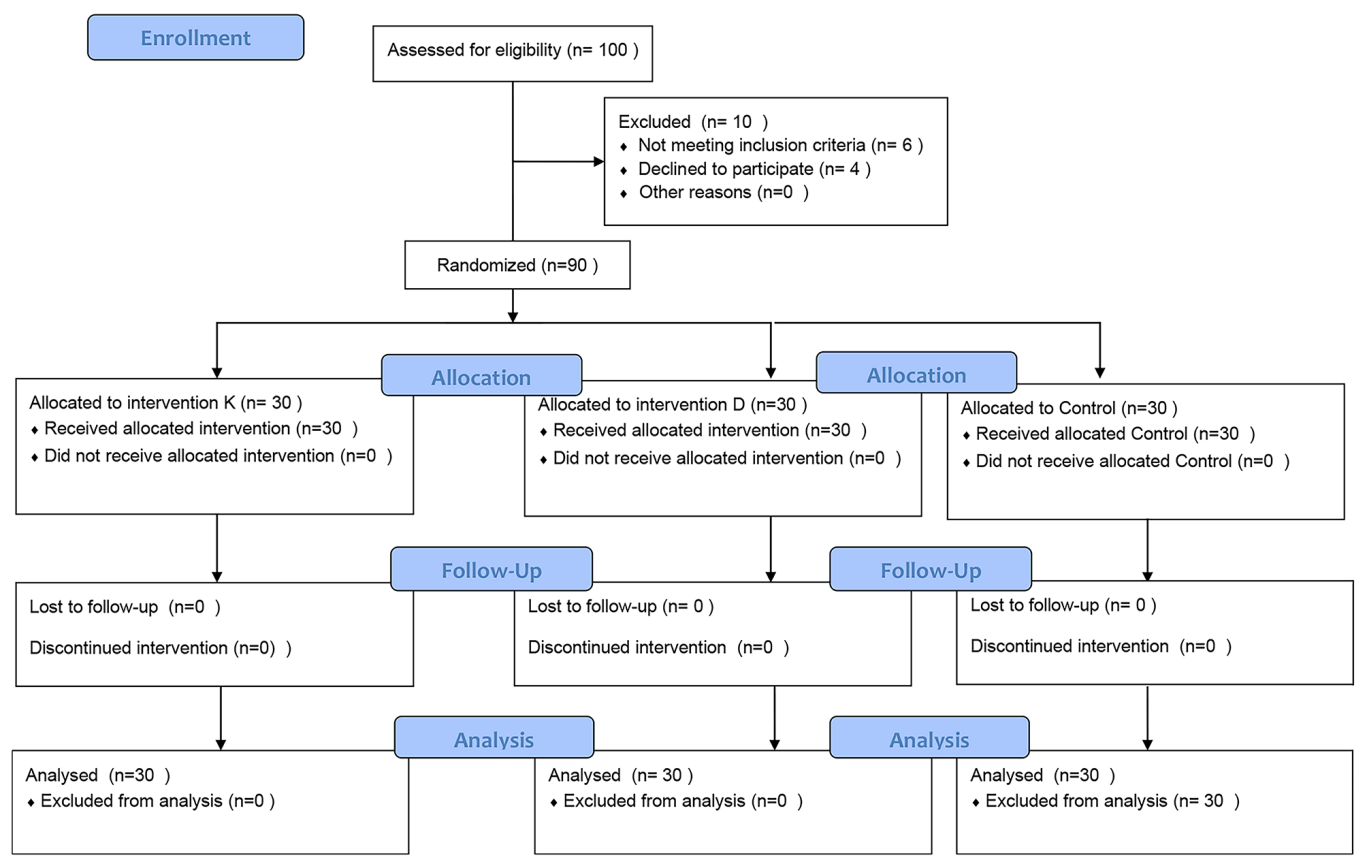
Flowchart of the study according to Consolidated Standards of Reporting Trials criteria (CONSORT).

A total of 100 patients were assessed for eligibility between April 2021 and November 2021. Ninety patients were enrolled in the study (30 in each group). Ten patients were excluded because of refusal to participate (four cases), a history of psychiatric disorders (three cases), and uncontrolled medical conditions (DM [two cases] and HTN [one case]) (Fig. 1).

There were no significant differences in the sex ratio, medical conditions, and BMI among the three groups (Table 3). The length of surgery was non-significantly higher in the control group than in the other groups (Table 4).

**Table 3:** Demographic data and preoperative medical conditions of the patients in the study groups

	Ketamine	Dexmedetomidine	Control	P-value
Sex - Male - Female	13 (43.33%) 17 (56.67%)	14 (46.67%) 16 (53.33%)	12 (40.00%) 18 (60.00%)	0.873
Age	37 ± 4	34 ± 7	34 ± 7	0.118
BMI (kg/m^2^)	41 ± 2	43 ± 4	43 ± 4	0.127
DM	13 (43%)	13 (43%)	17 (57%)	0.490
HTN	10 (33%)	7 (23%)	14 (47%)	0.162
Hypothyroidism	5 (17%)	4 (13%)	2 (6%)	0.484

BMI, body mass index; DM, diabetes mellitus; HTN, hypertension

Data are expressed as mean ± standard deviation or as numbers (percentages). There were no statistically significant differences in the above parameters between the three groups

**Table 4:** Length of surgery, time to extubation, and intraoperative total fentanyl doses in the study groups

		Mean	SD	P-value
Length of surgery (min)	Ketamine	87	15	0.128
	Dexmedetomidine	81	15	
	Control	88	17	
Time to extubation (min)	Ketamine	4	1	0.005
	Dexmedetomidine	3*	1	
	Control	4	1	
Intraoperative total fentanyl dose (µg)	Ketamine	160	42	< 0.001
	Dexmedetomidine	135*	37	
	Control	187	56	

SD, standard deviation

There were no statistically significant differences in the length of surgery between the three groups

* Compared with the other treatments, dexmedetomidine significantly reduced the time to extubation and intraoperative fentanyl requirement

**Fig. 2:**
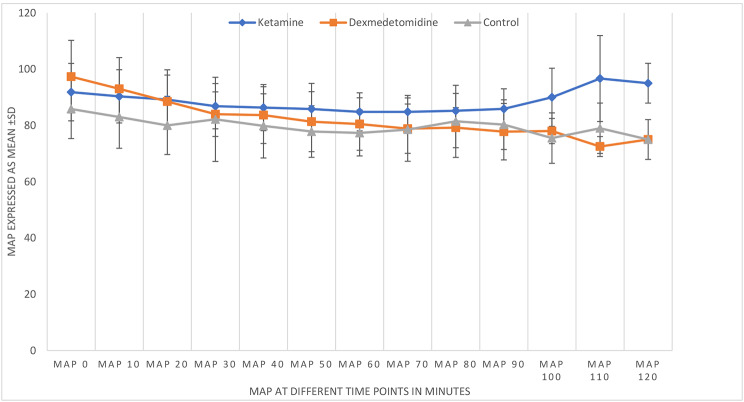
Mean arterial pressure values in the study groups MAP: mean arterial pressure

**Fig. 3:**
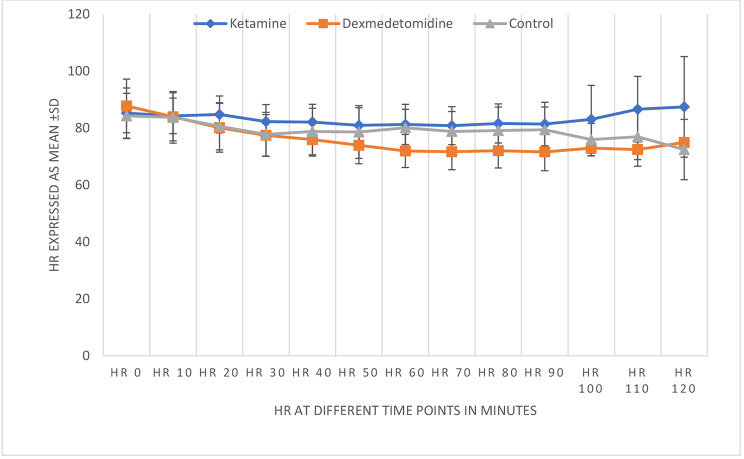
Heart rate values between the groups HR: heart rate

In terms of vital data, MAP values were higher in the ketamine group. There was no significant difference in the MAP between the dexmedetomidine group and the control group (Fig. 2). In turn, HRs were significantly lower in the dexmedetomidine group than in the other groups (Fig. 3).

The intraoperative total fentanyl doses were significantly lower in the dexmedetomidine group than in the other groups (Table 4). The mean fentanyl dose was marginally lower in the ketamine group than in the control group. Time to extubation was significantly shorter in the dexmedetomidine group than in the other groups (Table 4).

MOASS scores were better in the dexmedetomidine group than in the other groups, especially in the first 10 min (Table 5) and returned to baseline at 30 and 60 min in all groups.

**Table 5:** Modified observer’s assessment of alertness/sedation scales in the study groups

	Ketamine	Dexmedetomidine	Control	P-value
	Median (Q1–Q3)	Median (Q1–Q3)	Median (Q1–Q3)	
MOASS 0 min	4 (3–4)	4 (4–5)^‡^	3 (3–4)	0.017
MOASS 10 min	4 (4–5)	5 (5–5)^‡^	4 (4–5)	0.001
MOASS 30 min	5 (5–5)	5 (5–5)	5 (5–5)	0.415
MOASS 60 min	5 (5–5)	5 (5–5)	5 (5–5)	-

MOASS: modified observer’s assessment of alertness/sedation scale.

^‡^ Statistically significant difference between the dexmedetomidine group and the other groups.

NRS scores were significantly lower in the ketamine group than in the other groups (Table 6), reflected in lower morphine consumption (Table 7). Concerning the primary end-point of the study, significantly less cumulative morphine consumption postoperatively was observed in the ketamine group compared with the other two groups (p < 0.001).

**Table 6:** Numerical Rating Scale scores and PONV in the three different groups

	Ketamine	Dexmedetomidine		Control		P-Value
	Median (Q1–Q3)	Median (Q1–Q3)		Median (Q1–Q3)		
NRS 0 min	0 (0–1)^‡^	1(1–2)		2 (1–2)		< 0.001
NRS 30 minutes	1 (1–1)^‡^	2 (1–2.75)		2 (1.25–3)		< 0.001
NRS 60 minutes	1.5 (1–2)^†^	2 (2–3)		2 (2–4)		0.017
NRS 2 h	2 (2–3)	3 (2–4)		3 (2–5)		0.070
NRS 6 hours	3 (2–4)	3 (2–3.75)		3 (2–5)		0.726
NRS 12 hours	2 (2–3)^†^	3 (2–4)		3 (2–4)		0.012
NRS 24 hours	2 (1–3)	2.5 (2–3)		2 (2–3)		0.068
Nausea and Vomiting	13/30	8/30		16/30		0.106

NRS, Numerical Rating Scale. PONV, Postoperative nausea and vomiting

‡ Statistically significant difference between the ketamine group and the two other groups

† Statistically significant difference between the ketamine group and the dexmedetomidine group. Statistically non-significant results between the control and other groups

**Table 7:** Total morphine consumption postoperatively in the study groups

Total morphine consumption postoperatively (mg)		Mean	SD	P-value
	Ketamine	3*	3	< 0.001
	Dexmedetomidine	5	4	
	Control	7	3	

SD, standard deviation

*Statistically significant difference between the ketamine group and the other two groups

PONV scores were lower in the dexmedetomidine group than in the other groups (Table 6). There were no severe postoperative complications, such as airway obstruction, life-threatening hypoxic events, or severe PONV.

## Discussion

Herein, we compared the analgesic effect of ketamine and dexmedetomidine during bariatric surgery on postoperative total morphine consumption and found that ketamine significantly reduces the total morphine consumption postoperatively and dexmedetomine significantly reduces intraoperative total consumption of fentanyl.

Around half a million treatments for obesity are performed each year globally. Most of these procedures are performed under laparoscopy [15] and are associated with high levels of postoperative pain [16]. With multiple system changes and a high burden of comorbidities, patients with morbid obesity (MO) are at increased risk of morbidity and adverse effects during pain management [17]. Thus, these patients require a more comprehensive approach to pain management [17]. In addition, current literature supports the use of systemic analgesics and highlights the need for further research in the pharmacological and non-pharmacological management of acute pain in these patients [17].

Our study showed lower intraoperative fentanyl doses, a shorter time to extubation, and improved MOASS and PONV scores which were all linked to dexmedetomidine infusion. Significantly lower NRS scores and postoperative morphine doses were linked to ketamine infusion. These findings showed that while ketamine reduced the need for morphine, dexmedetomidine effectively reduced the intraoperative fentanyl required and the time to extubation. Compared to dexmedetomidine, ketamine infusion minimizes the requirement for opioids for postoperative pain control with little side effects. Our findings were similar to those of Garg et al., who demonstrated that ketamine and dexmedetomidine provide good and safe analgesia, decrease the need for morphine, and increase the pain-free period during the postoperative period in patients undergoing spinal surgery [13].The same regimens were used to evaluate the effect of these drugs on obese patients undergoing bariatric surgeries.

In our cohort, demographic data were similar among the three groups. The most common diseases were DM and HTN, which are correlated with obesity as components of metabolic syndrome [18]. The second most common disease was hypothyroidism, which may contribute to obesity [19].

Intraoperative MAP tended to be higher in the ketamine group than in the other groups. Conversely, HR values were similar between the ketamine group and control group, suggesting that the sympathomimetic action of ketamine does not preclude an analgesic effect. HR values were significantly lower in the dexmedetomidine group, probably because of decreased sympathetic outflow, and this finding supports the analgesic effect of this drug. Blood pressure and HR did not decrease significantly across the groups, suggesting that both drugs can maintain stable hemodynamics during surgery.

In our cohort, the need for analgesics intraoperatively was significantly lower in the dexmedetomidine group than in the other groups. Further, ketamine group was associated with a lower fentanyl requirement than control group. Consistent with our finding, Bakhamees et al. found that dexmedetomidine decreased the need for fentanyl intraoperatively in patients with MO undergoing laparoscopic bypass surgery [20]. Conversely, Ali et al. observed that ketamine failed to decrease fentanyl consumption intraoperatively in obese patients undergoing abdominoplasty surgery. A study conducted by Seman et al. showed ketamine reduced fentanyl consumption intraoperatively [21]. This discrepancy may be due to differences in infusion regimens across studies [22].

Dexmedetomidine marginally decreased the time to extubation and marginally improved MOASS scores. Alertness returned to normal within 60 min postoperatively in all groups. There were no cases of emergencies or agitation during recovery from anesthesia. Consistent with our results, Zeeni et al. observed that dexmedetomidine and morphine infusions administered during laparoscopic bariatric surgery led to the same level of postoperative sedation [23]. Furthermore, Tufanogullari et al. found no significant differences in postoperative sedation and time to extubation among three dexmedetomidine regimens and controls [24]. Similar to our study, Ali et al. found no significant difference in postoperative sedation between ketamine and morphine infusion [22].

NRS scores correlated with the need for morphine as rescue analgesia in the postoperative period. NRS scores were significantly lower in the ketamine group at most time points. Our primary outcome was the effect of ketamine and dexmedetomidine on total morphine requirement 24 h post-surgery. The need for morphine was lowest in the ketamine group (mean: 3 ± 3 mg). Further, the need for morphine was marginally lower in the dexmedetomidine group than in control group; however, the benefits of reduced postoperative morphine requirement cannot be ignored. Similar to our results, Mehta et al. found that ketamine infusion decreased postoperative analgesic requirements compared with a control in patients undergoing laparoscopic bariatric surgery [25].

In terms of postoperative pain control, ketamine can also help reduce the development of chronic post-operative pain, in addition to its opioid-relieving effect. This can be achieved through the inhibition of the receptors of the N-methyl-D-aspartate and by reduction of wind-up and central sensitization [26]. The analgesic effect of dexmedetomidine is mainly mediated by the α_2c_ and α_2a_ receptors located in the dorsal horn by inhibiting the pro-nociceptive transmitters, primarily substance P and glutamate, and the hyperpolarization of the spinal interneurons [27].

Our results demonstrated that dexmedetomidine infusion reduced PONV scores, suggesting that this drug had an anti-emetic effect during the postoperative course. Hussein and Mostafa showed that dexmedetomidine reduced PONV in patients undergoing bariatric surgery compared with controls [28]. In addition, Brinck et al. showed that perioperative IV ketamine could potentially reduce PONV. Ketamine marginally decreased PONV scores, and this decrease might have clinical significance [26]. A noteworthy finding is that no serious postoperative complications were recorded in any of the patients in our study.

For these reasons, we suggest that ketamine or dexmedetomidine should be considered as part of multidisciplinary therapy for all patients with chronic diseases or with chronic pain syndromes undergoing painful surgeries. Future long-term studies are essential to address the issue of prevention of postoperative chronic pain syndromes. Additionally, we suggest that the effects of ketamine and dexmedetomidine in patients that are at high risk for developing acute postoperative morphine-resistant pain (cancer patients, chronic pain syndromes, amputation, and drug dependence) should be investigated. Combined effect of both drugs is a point of future study.

## Limitations

Confounding factors that might affect the need for analgesia, such as depression and pre-existing pain, were not assessed preoperatively. Thus, a long-term assessment of the effect of these factors on postoperative pain is needed. The lack of long term assessment of pain scores is also a limitation of our study.

## Conclusion

Ketamine and dexmedetomidine can effectively manage pain in patients with MO undergoing bariatric surgery. Dexmedetomidine improves clinical outcomes in the intraoperative period, while ketamine improves analgesia in the postoperative period. In addition, dexmedetomidine may improve postoperative recovery in terms of sedation and PONV.

## Electronic supplementary material

Below is the link to the electronic supplementary material.

Supplementary Material 1

## Data Availability

The data collected during the study from deindividualized participants will be shared with researchers. Materials including the study protocol, informed consent forms, and statistical analysis plan will be made available for use after a review of the study’s methodology. Request for access to patient data should be sent to the corresponding author. The study management committee will then review the requests and determine if the authors should be granted access to the data. To gain access to the data, researchers must first sign a data access agreement. This agreement should specify that the data will only be used for the purpose that was agreed upon.

## Abbreviations

ACE: angiotensin converting enzyme
BMI: body mass index
CCB: calcium channel blocker
DM: diabetes mellitus
HR: heart rate
HTN: hypertension
IBW: ideal body weight
IV: intravenous
LBW: lean body weight
MAP: mean arterial pressure
MO: morbid obesity
MOASS: modified observer’s agitation/sedation scale
NRS: numerical rating scale
PACU: post anesthesia care unit
PONV: postoperative nausea and vomiting
SGLUT: Sodium-dependent glucose cotransporter
TBW: total body weight
